# A seasonal switch in histone deacetylase gene expression in the hypothalamus and their capacity to modulate nuclear signaling pathways

**DOI:** 10.1016/j.bbi.2016.12.013

**Published:** 2017-03

**Authors:** Patrick N. Stoney, Diana Rodrigues, Gisela Helfer, Thabat Khatib, Anna Ashton, Elizabeth A. Hay, Robert Starr, Dagmara Kociszewska, Peter Morgan, Peter McCaffery

**Affiliations:** a Institute of Medical Sciences, University of Aberdeen, Foresterhill, Aberdeen AB25 2ZD, Scotland, UK; b Rowett Institute of Nutrition and Health, University of Aberdeen, Greenburn Road, Bucksburn, Aberdeen AB21 9SB, Scotland, UK; c Faculty of Life Sciences, University of Bradford, Richmond Road, Bradford BD7 1DP, UK

**Keywords:** AP-1, activator protein-1, Dio, deiodinase, Bdnf, brain derived neurotrophic factor, cAMP, cyclic adenosine monophosphate, DMEM, Dulbecco’s modified eagle medium, GFAP, glial fibrillary acidic protein, HDAC, histone deacetylase, HSP90, heat-shock protein 90, Iba1, ionized calcium-binding adapter molecule 1, LCoR, ligand-dependent corepressor, LD, long-day, LPS, lipopolysaccharide, MAP2, microtubule-associated protein 2, Nrgn, neurogranin, NF-κB, nuclear factor kappa-light-chain-enhancer of activated B cells, Prkc, protein kinase C, PBS, phosphate-buffered saline, Ptp1b, protein-tyrosine phosphatase 1B, qPCR, quantitative polymerase chain reaction, RAR α, β and γ, retinoic acid receptor alpha, beta and gamma, RARE, retinoic acid response element, SD, short-day, T3, triiodothyronine, THR, thyroid hormone receptor, TNFα, tumour necrosis factor alpha, TRE, thyroid hormone response element, TSH, thyroid-stimulating hormone, Histone deacetylase, Nucleus, Inflammatory, Tumour necrosis factor alpha, Lipopolysaccharide

## Abstract

Seasonal animals undergo changes in physiology and behavior between summer and winter conditions. These changes are in part driven by a switch in a series of hypothalamic genes under transcriptional control by hormones and, of recent interest, inflammatory factors. Crucial to the control of transcription are histone deacetylases (HDACs), generally acting to repress transcription by local histone modification. Seasonal changes in hypothalamic HDAC transcripts were investigated in photoperiod-sensitive F344 rats by altering the day-length (photoperiod). HDAC4, 6 and 9 were found to change in expression. The potential influence of HDACs on two hypothalamic signaling pathways that regulate transcription, inflammatory and nuclear receptor signaling, was investigated. For inflammatory signaling the focus was on NF-κB because of the novel finding made that its expression is seasonally regulated in the rat hypothalamus. For nuclear receptor signaling it was discovered that expression of retinoic acid receptor beta was regulated seasonally. HDAC modulation of NF-κB-induced pathways was examined in a hypothalamic neuronal cell line and primary hypothalamic tanycytes. HDAC4/5/6 inhibition altered the control of gene expression (*Fos*, *Prkca*, *Prkcd* and *Ptp1b*) by inducers of NF-κB that activate inflammation. These inhibitors also modified the action of nuclear receptor ligands thyroid hormone and retinoic acid. Thus seasonal changes in HDAC4 and 6 have the potential to epigenetically modify multiple gene regulatory pathways in the hypothalamus that could act to limit inflammatory pathways in the hypothalamus during long-day summer-like conditions.

## Highlights

• Seasonal changes in rat trigger change in hypothalamic histone deacetylases (HDACs).
• NF-κB is an inflammatory regulator under seasonal control in the hypothalamus.
• These HDACs may control hypothalamic inflammatory and nuclear receptor pathways.

## 1 Introduction

There is a growing interest in the regulation of hypothalamic function by epigenetic mechanisms – control of gene expression via chemical modifications of DNA or chromatin (Gali Ramamoorthy et al., 2015). Histone deacetylases (HDACs) are essential mediators of epigenetic regulation and act by removing acetyl groups from lysine residues of histones, leading to chromatin condensation and thus transcriptional repression. The HDACs fall into four groups, primarily based on homology to yeast equivalent genes: class I (HDACs1, 2, 3 and 8), class IIa (HDACs 4, 5, 7 and 9), class IIb (HDACs 6 and 10), and class IV, consisting of HDAC11 alone. Two HDACs of particular relevance to this study, HDACs 4 and 6, have more complex functions than simply deacetylating histones.

HDAC4 can move between the nucleus and cytoplasm, with functions in both subcellular compartments (Fitzsimons, 2015). It is associated with disorders such as 2q37-deletion syndrome which includes facial dysmorphism, brachydactyly and obesity with reduced expression of the *RAI1* gene (Williams et al., 2010). HDAC6 also shuttles between the nucleus and cytoplasm (Liu et al., 2012) and it is associated with a variety of disorders from cancer to neurodegenerative disease (Seidel et al., 2015). Several putative functions for HDAC6 in the nucleus have been described (reviewed by Yang and Gregoire, 2005), but HDAC6 has been extensively investigated as an alpha-tubulin deacetylase in the cytoplasm, regulating microtubule stability and cell motility (Hubbert et al., 2002) during development (Creppe et al., 2009). In neurodegenerative disease, HDAC6 may increase autophagy to protect neurons from an accumulation of misfolded protein caused by impairment of the ubiquitin-proteasome system (Pandey et al., 2007). In addition, HDAC6 associates directly with ubiquitin and the binding of HDAC6 to polyubiquitinated proteins increases expression of cellular chaperones and protects against the harmful effects of misfolded proteins (Boyault et al., 2007).

This study hypothesized that there would be an association between day length (photoperiod) and HDAC gene expression levels in the hypothalamus of animals that respond to seasonal change. Many animals change their physiology and behavior between seasons, recognizing the seasonal difference in day length; the hypothalamus is the brain region in central control of these changes (Ebling, 2015). Animal models such as the photoperiod-sensitive F344 rat (Heideman and Sylvester, 1997) can be used to study the shift in feeding and body weight in response to laboratory-controlled alterations in day length. The expression of *Hdacs* 1–11 was compared in F344 rats maintained under long-day (16 h light:8 h dark) and short-day (8 h light:16 h dark) conditions. Of the *Hdacs* examined, only *Hdac4* and *Hdac6,* and to a lesser extent *Hdac9,* were photoperiodically regulated in the hypothalamus, with higher expression under long-day photoperiod relative to short-day. A series of studies using inhibitors of these HDACs suggested that one of their functions is the control of gene expression and this has the capacity to modulate inflammatory and hormone (thyroid hormone and retinoic acid) signaling pathways in the hypothalamus.

## 2 Materials and methods

### 2.1 Animals

Sprague Dawley rats were bred in the University of Aberdeen animal facility and kept in a 12 h:12 h light:dark cycle with unlimited access to food and water. Male F344/NHsd rats were supplied by Harlan Sprague-Dawley Inc. at 5–6 weeks of age and acclimatized for approximately 14 days in 12 h:12 h light:dark. For photoperiodic analysis of gene expression, F344 rats were randomly divided into two weight-matched groups of 7 each and transferred to either short day (SD; 8 h light:16 h dark) or long day (LD; 16 h light:8 h dark) photoperiod. For *in situ* hybridization and qPCR, F344 rats were anesthetized with isoflurane after 28 days in photoperiod and killed by decapitation. The brains were removed, rapidly frozen on dry ice and stored at −80°C. For immunohistochemistry, F344 rats were anesthetized with isoflurane after 28 days in LD photoperiod and transcardially perfused with 4% paraformaldehyde in phosphate buffer. The brains were then infused with 30% sucrose at 4°C, frozen and stored at −80°C.

F344 rats used for central thyroid-stimulating hormone (TSH) infusions were also used for gene expression analysis reported in another study and are fully described in Helfer et al. (2013). All procedures conformed to Home Office regulations and local ethics committee guidelines.

### 2.2 Hypothalamic organotypic slice cultures

*Ex vivo* hypothalamic slice cultures were set up as previously described in detail (Stoney et al., 2016b). Briefly, 400 μm-thick coronal hypothalamic slices were prepared from P10-12 male Sprague Dawley rat pups and maintained *ex vivo* on Millicell-CM cell culture inserts (Millipore) in serum-free, vitamin A-deficient medium consisting of Neurobasal medium containing B27 supplement without vitamin A, penicillin-streptomycin and Glutamax (all reagents from Invitrogen) and 5 mg/ml additional glucose. Before plating out, the slices were cut in half along the midline, giving two sets of slices per animal, with each containing the same (but alternate) regions. After 3 days in serum-free medium, slices were treated with 10 mIU bovine thyroid-stimulating hormone (TSH; Sigma Aldrich) dissolved in phosphate-buffered saline (PBS). One set of slices from each animal was treated with the other set being used as control. After 48 h of treatment, slices were excised from the culture inserts and frozen rapidly on dry ice for RNA extraction.

### 2.3 Immunohistochemistry

40 μm-thick coronal sections from F344 rat brains were cut using a cryostat. Sections containing the hypothalamus were used for immunohistochemistry as previously described (Shearer et al., 2012a, Stoney et al., 2016a). Sections were labelled with antibodies against HDAC4 (ab79521, Abcam), MAP2 (ab5392, Abcam), GFAP (G3893, Sigma-Aldrich), Iba1 (ab5076, Abcam) and vimentin (V6389, Sigma-Aldrich).

### 2.4 Cell culture

Primary tanycyte cultures were prepared from 10-day-old male Sprague Dawley rat pups as previously described (Bolborea et al., 2015, De Seranno et al., 2004, Prevot et al., 2003). Briefly, brains were removed under sterile conditions, placed in ice-cold DMEM/F-12 medium containing penicillin/streptomycin and 25 mM HEPES and the median eminence dissected. Median eminences from 8–10 pups were pooled together, dissociated with trypsin and plated out in DMEM/F-12 containing 10% fetal calf serum and penicillin/streptomycin in a 25 cm^2^ cell culture flask. The medium was replaced every 2–3 days and the cells transferred to 12-well plates for experiments after 8–10 days *in vitro*. The day after plating out, tanycytes were treated with vehicle or HDAC inhibitor (Tubastatin A at 5 μM or LMK235 at 1 μM) for 1 h and then LPS at 1 μg/ml for 3 h. RNA was extracted from treated cells for qPCR analysis.

The cell line GT1-7 (Mellon et al., 1990) is a widely used hypothalamic cell line used in over 400 published papers. GT1-7 cells were maintained in Dulbecco’s modified eagle medium with 10% fetal bovine serum. To examine the influence of HDACs on TNFα function the cells were treated with vehicle or HDAC inhibitor (Tubastatin A at 5 μM or LMK235 at 1 μM) for 1 h and then TNFα at 1 ng/mL for 3 h. Alternatively, to investigate the influence of HDACs on retinoic acid or T3 function, the cells were treated with retinoic acid (1 μM) or T3 (50 nM) in addition to an HDAC inhibitor (Tubastatin A at 5 μM or LMK235 at 1 μM) or vehicle for 24 h. In both cases RNA was extracted from treated cells for qPCR analysis.

### 2.5 Immunocytochemistry

Primary tanycytes and GT1-7 cells were plated onto polylysine-coated glass coverslips. The following day, the cells were fixed and immunolabelled as previously described (Shearer et al., 2012a) using antibodies against HDAC4 (as above), HDAC6 (ab1440, Abcam) and NeuN (MAB377, Chemicon).

### 2.6 Quantitative polymerase chain reaction (qPCR)

Hypothalamic blocks encompassing the mediobasal hypothalamus were dissected from F344 brains whilst frozen and total RNA was extracted using a Qiagen RNeasy RNA purification kit. RNA was isolated from cultured hypothalamic slices using the same kit. cDNA was synthesized from 500 ng total RNA using High Capacity RNA-to-cDNA Master Mix (Applied Biosystems Ltd). Primers were designed using PrimerBLAST (Ye et al., 2012; Table 1), apart from *Dio2* and *Dio3* primers, which were obtained from Qiagen (QuantiTect Primer Assays Rn_Dio2_2_SG and Rn_Dio3_1_SG, respectively). qPCR reactions were set up using SensiMix SYBR master mix (Bioline) and were run on a Roche LightCycler 480 and analyzed using LightCycler 480 1.5 software. Expression of genes of interest was normalized to *Actb* levels. Standard curves and blank controls were run for all sets of primers. Differences in gene expression were assessed by Student’s *t*-test or ANOVA as appropriate.

### 2.7 *In situ* hybridization

*In situ* hybridization was performed as described in detail in (Ross et al., 2009). A riboprobe template for *Hdac4* was amplified by PCR from rat hypothalamic cDNA using forward 5′-CAG CCC TCC AGC AGC GAA TCT CC-3′ and reverse 5′-TCT GTC TCC TCC GGG TGG CTC TCA-3′ primers and cloned into pCR4 Blunt TOPO vector (Invitrogen). For *Nfkb1* an EST clone was purchased from Source BioSciences (IMAGE ID: 7366143). ^35^S-labelled antisense riboprobes were generated from 150–200 ng template DNA by T7 polymerase. Slides were apposed to Biomax MR Film (Kodak) for 7 days, scanned at 1200 dpi and analyzed using Image-Pro Analyzer (Media Cybernetics UK).

### 2.8 Western blotting analysis

Cells for analysis were washed with cold PBS and lysed with cell lysis buffer (150 mM NaCl, 1% Triton, 0.1% SDS, 50 mM HEPES) containing protease inhibitor cocktail (Sigma). Protein concentrations were measured using a BCA assay kit (ThermoFisher Scientific). 50 μg protein was loaded and separated on 12% SDS-polyacrylamide gels and then transferred to nitrocellulose membranes. The membranes were blocked with 5% milk in Tris-buffered saline containing 0.05% Tween-20 (TBST) for 1 h at room temperature. The blots were incubated overnight in primary antibody at 4°C diluted in TBST containing 2% BSA. The following day, the blots were washed three times with TBST before incubating with horseradish peroxidase-conjugated antibody (Jackson Immunoresearch) diluted in TBST containing 5% milk for 1 h at room temperature. Finally, after 3 washes in TBST, the blots were developed using enhanced chemiluminescence (Millipore) and the protein bands were detected and scanned using a myECL Imager (ThermoScientific). Afterwards, band intensities were quantified from images using ImageJ software. Antibodies used were against: α-tubulin (T6074, Sigma), acetylated α-tubulin (Lys40, 5335, NEB), NF-κB (Cell Signaling, 4764), and phospho-NF-κB p65 (Ser536, Cell Signaling, 3033).

## 3 Results

### 3.1 *Hdac* gene expression in the rat hypothalamus switches between seasons

The seasonal changes in animals that influence behaviors such as appetite and reproduction are regulated by the hypothalamus using a variety of signaling routes that regulate gene expression. Nuclear receptor family members are important mediators of such signaling pathways (Ebling, 2014, Ebling, 2015) and HDACs, by controlling the open or closed state of chromatin, can have a profound influence on their action. The expression of HDACs 1–11 was investigated in animals kept for 28 days under long-day (LD, 16 h:8 h light:dark) or short-day (SD, 8 h:16 h light:dark) conditions.

**Fig. 1:**
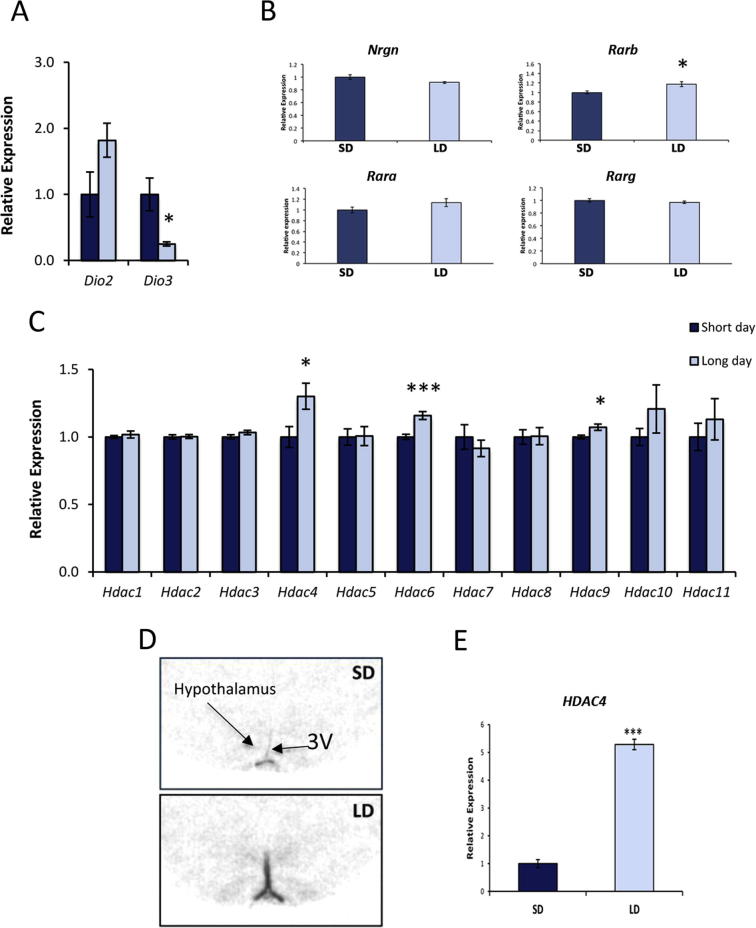
Comparison between short-day versus long-day photoperiod reveals changes in *Hdac* gene expression in the hypothalamus. (A) As positive controls, hypothalamic expression of *Dio2* increased and *Dio3* decreased in long-day conditions relative to short-day. (B) A reporter of thyroid hormone signaling (*Nrgn*) did not change between short- and long-day while a reporter of retinoic acid signaling (*Rarb*) significantly increased in long-day conditions but no such change was seen for other members of the RAR family, *Rara* or *Rarg*. (C) Three HDAC genes, *Hdac4*, *6* and *9* showed significantly higher expression in long-day photoperiod, although the change in *Hdac9* was small. (D) *In situ* hybridization verified the increase in expression of *Hdac4* predominantly in the ependymal layer around the third ventricle (3V). (E) Quantification of *in situ* hybridization in the ependymal layer shows a significant five-fold increase in *Hdac4* expression in long-day photoperiod. qPCR quantification of gene expression is shown relative to *Actb* levels. ^∗^p < 0.05, ^∗∗∗^p < 0.001.

As positive controls for a response to the change in light duration, we measured the expression of *Dio2* and *Dio3*, which encode the deiodinase enzymes that respectively activate (Dio2) or inactivate (Dio3) thyroid hormone. Consistent with previous studies were the reciprocal changes in *Dio2* and *Dio3* expression following a LD to SD switch (Helfer et al., 2013, Watanabe et al., 2004, Watanabe et al., 2007), although only the change in *Dio3* was statistically significant in the current study (p = 0.013; Fig. 1A). As further controls for the known LD increase in thyroid hormone (Dardente et al., 2014) and retinoic acid signaling (Shearer et al., 2012b), expression of *Nrgn*, a gene with a well-characterized thyroid hormone response element (Martinez de Arrieta et al., 1999) and *Rarb*, with a well-characterized retinoic acid response element (de Thé et al., 1990) were quantified. No significant change in *Nrgn* was evident (Fig. 1B) but the rise in retinoic acid signaling was sufficient to induce *Rarb* but not family members *Rara* or *Rarg* (Fig. 1B). Of the HDACs, only *Hdac4, 6* and *9* showed significant differences in expression between LD and SD photoperiod (p = 0.035, p = 0.001, p = 0.022, respectively; Fig. 1C). Focusing on the HDAC with the largest change in expression by qPCR, the distribution of *Hdac4* was examined by *in situ* hybridization after 28 days in LD photoperiod. Strong expression was evident in the ependymal layer lining the third ventricle (Fig. 1D), a region that includes tanycytes, known to be important regulators of appetite and energy balance in the hypothalamus (Bolborea and Dale, 2013). Lower expression of *Hdac4* was also apparent in the parenchyma of the hypothalamus (Fig. 1D). Quantifying expression in the lining of the third ventricle, the expression of *Hdac4* was approximately 5-fold higher in LD compared to SD (p < 0.001; Fig. 1E). This suggests that, although qPCR analysis of the entire hypothalamus provides a useful method for screening (Fig. 1C), it underestimates the changes in expression between photoperiod in local regions where expression changes are large.

**Fig. 2:**
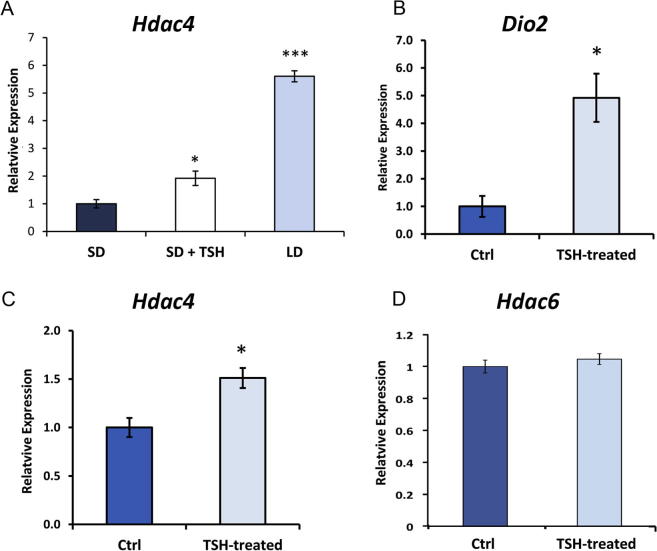
Regulation of *Hdac4* expression in the hypothalamus by thyroid-stimulating hormone (TSH) . (A) Infusion of TSH into the third ventricle of the short-day rat induced *Hdac4* expression and so partially mimicked the effect of long-day conditions. (B) The direct effect of TSH on the hypothalamus could be demonstrated by the addition of TSH to hypothalamic organotypic slice cultures which induced *Dio2* as a positive control. (C) *Hdac4* also responded to TSH in hypothalamic organotypic slice cultures whereas (D) *Hdac6* did not. qPCR quantification of gene expression is shown relative to *Actb* levels. ^∗^p < 0.05, ^∗∗∗^p < 0.001.

Thyroid-stimulating hormone (TSH) acts as an essential intermediary in the control of seasonal changes in the hypothalamus and drives photoperiodic changes in gene expression (Dardente et al., 2014). To determine whether TSH may be a trigger to induce *Hdac4* expression, animals were maintained under SD conditions when *Hdac4* expression is at its lowest and TSH or vehicle was infused into the third ventricle. Fourteen days of central TSH infusion (1.0 mIU/day) resulted in a significant increase in *Hdac4* expression (p = 0.035; Fig 2A) although this did not reach the same level of expression seen under LD conditions. To confirm that TSH could act directly on the hypothalamus to induce *Hdac4* expression, organotypic slice cultures of the hypothalamus were treated with 10 mIU of TSH. It was confirmed that TSH in this system significantly induced *Dio2* (Shinomiya et al., 2014) (p = 0.015, Fig 2B), while the expected decline in *Dio3* was reported previously (Stoney et al., 2016b). As was already demonstrated *in vivo,* TSH also significantly induced *Hdac4* expression in organotypic hypothalamic slice cultures (p = 0.023; Fig. 2C). In contrast, *Hdac6* was not induced by TSH (Fig. 2D).

### 3.2 HDAC4 and 6 are strongly expressed in the nucleus

**Fig. 3:**
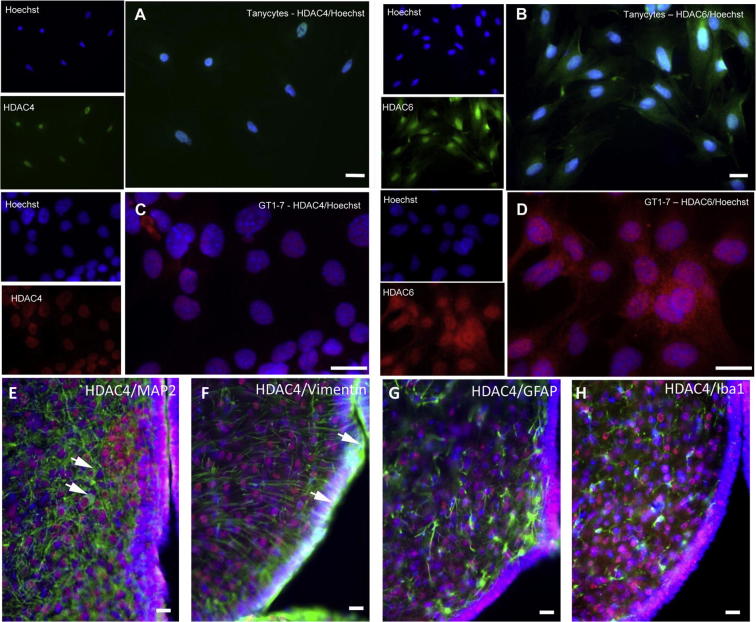
Immunohistochemistry of HDAC4 and HDAC6 in hypothalamic cells shows their expression in the nucleus. (A) HDAC4 in cultured tanycytes and (C) the hypothalamic neuronal GT1-7 cell line showed that it is present only in the nucleus. HDAC6 was localized to the nucleus of cultured tanycytes (B) and GT1-7 cells (D) but was also present at lower levels in the cytoplasm. (E-H) Double-labelling of rat brain sections, with nuclei stained blue with bisbenzamide, anti-HDAC4 in red and antibodies to the following markers in green: (E) MAP2 for neurons with two double-labelled cells highlighted with white arrows (F), vimentin for tanycytes with two double-labelled cells highlighted with white arrows, (G) GFAP for astrocytes and (H) Iba1 to label microglia. This illustrated that HDAC4 is present in both neurons and tanycytes but absent from microglia and glia. Scale bars = 25 μm.

**Fig. 4:**
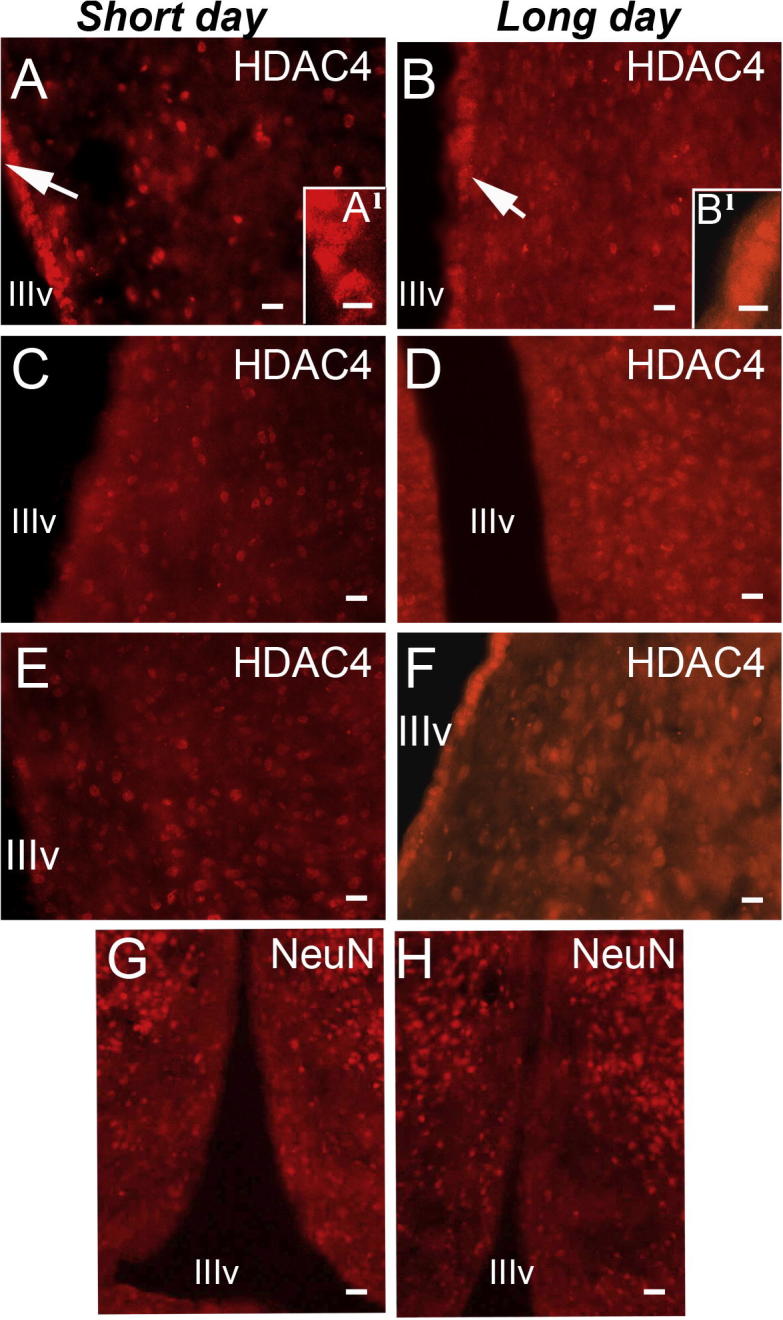
Expression of HDAC4 in ventral hypothalamus in long-day compared to short-day conditions. HDAC4 was expressed in both the ependymal cell layer (white arrow A and B) and the parenchyma in the hypothalamus of six rats kept in either (A, C, E) short-day or (B, D, F) long-day conditions. Variation in expression in the ependymal layer was quite high but HDAC4 was clearly present in nuclei, and more weakly in cytoplasm, in some ependymal cells in both (A^1^) short-day and (B^1^) long-day conditions, shown at higher magnification. Expression of HDAC4 though was generally stronger in long-day compared to short-day conditions while expression of (G and H) NeuN was similar in both conditions. Scale bars = 20 μm in A-F, 40 μm in G and H and 12 μm in A^1^ and B^1^.

The focus on the possible functions of HDACs in the hypothalamus was directed towards *Hdac4* and *Hdac6*, which showed the largest significant changes between photoperiods. The primary drive behind this study was to investigate HDACs as regulators of gene expression; however, both HDAC4 and 6 have major functions in the cytoplasm (Fitzsimons, 2015, Seidel et al., 2015). Nevertheless, several studies have suggested that they can act as conventional HDACs and regulate gene expression and this is dependent on their presence in the nucleus (Fitzsimons, 2015, Seidel et al., 2015). Nuclear versus cytoplasmic distribution of the HDACs was examined by immunocytochemistry in primary cultured tanycytes, TSH-responsive cells lining the third ventricle in which the *Hdac4* transcript was present (Fig. 1D), and the GT1-7 mouse hypothalamic neuronal cell line (Mellon et al., 1990). In both cell types, the distribution of HDAC4 was primarily nuclear, while HDAC6 was present in the nucleus and cytoplasm (Fig. 3A–D). Thus both HDAC4 and 6 have the capability to influence nuclear processes in these cell types. To determine the distribution of HDACs in the hypothalamus, immunohistochemistry was performed on brain sections from F344 rats kept on LD photoperiod for 28 days. Only the antibody against HDAC4 showed specific staining under these conditions and HDAC4 was predominantly found in the nucleus (Fig. 3E–H). To determine the cell types expressing HDAC4, rat brain sections were double-labelled with antibodies against HDAC4 and the cell type-specific markers MAP2 for neurons, vimentin for tanycytes, GFAP for astrocytes, and Iba1 to label microglia. HDAC4 was present in both neurons and the ependymal cell lining of the third ventricle, which includes tanycytes (Fig. 3E and F) but was absent from microglia and glia (Fig. 3G and H). Expression of HDAC4 was compared between brain sections from six rats after 28 days in either SD or LD photoperiod and Fig.4A–F shows images of the ventral hypothalamus in the region of the arcuate nucleus. HDAC4 expression in both SD (Fig. 4A, C, E) and LD (Fig. 4B, D, F) was observed in the ependymal cell layer as well as the parenchyma of the hypothalamus. Despite considerable variation in HDAC4 expression in the ependymal layer of different rats, there were no major changes in nuclear versus cytoplasmic HDAC4 expression in this region (Fig. 4A^1^ and B^1^). However, expression of HDAC4 appeared stronger in LD compared to SD conditions while expression of another protein, NeuN, which like HDAC4 is predominantly present in the nucleus and weakly present in the cytoplasm (Lind et al., 2005), showed similar expression in hypothalami between LD and SD conditions (Fig. 4G and H).

### 3.3 *Nfkb1* is an inflammatory-mediating gene in the hypothalamus that switches in expression between seasons

**Fig. 5:**
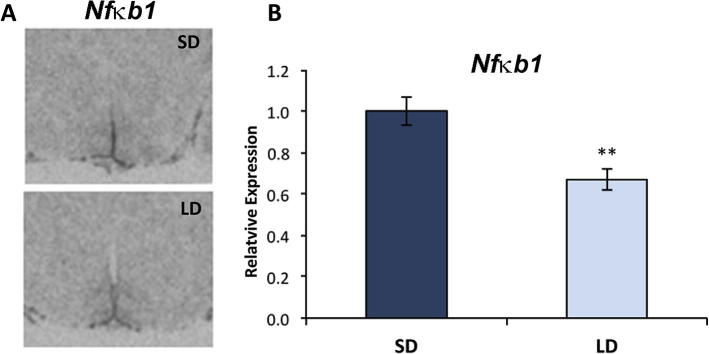
Seasonal change in daylength results in altered expression of the nuclear factor *Nfkb1* transcript. (A) *In situ* hybridization showed a decrease in *Nfkb1* expression in the cells lining the third ventricle of the hypothalamus with change in photoperiod from short- to long-day. (B) Quantification of this change indicated significantly lower expression in long-day photoperiod relative to short-day. qPCR quantification of gene expression is shown relative to *Actb* levels. ^∗∗^p < 0.01.

Due to their nuclear localization, we next explored the potential for HDAC4 and 6 to modulate pathways controlling gene expression. Previous studies of the role of HDACs in cell proliferation (Mottamal et al., 2015) have pointed to a central mechanism of HDAC action through the nuclear transcription factor nuclear factor kappa-light-chain-enhancer of activated B cells (NF-κB; (Glozak and Seto, 2007)). Therefore, the effect of photoperiod on NF-κB signaling was investigated. *In situ* hybridization showed that *Nfkb1* gene expression is significantly altered in the hypothalamus with photoperiod showing the reverse pattern to the HDACs with lower levels under LD versus SD photoperiod conditions (p = 0.003; Fig. 5A and B). Its expression is predominantly in the ependymal lining of the third ventricle of the hypothalamus, similar to that of *Hdac4*.

### 3.4 Inhibition of HDACs modifies gene expression induced by NF-κB activators

**Fig. 6:**
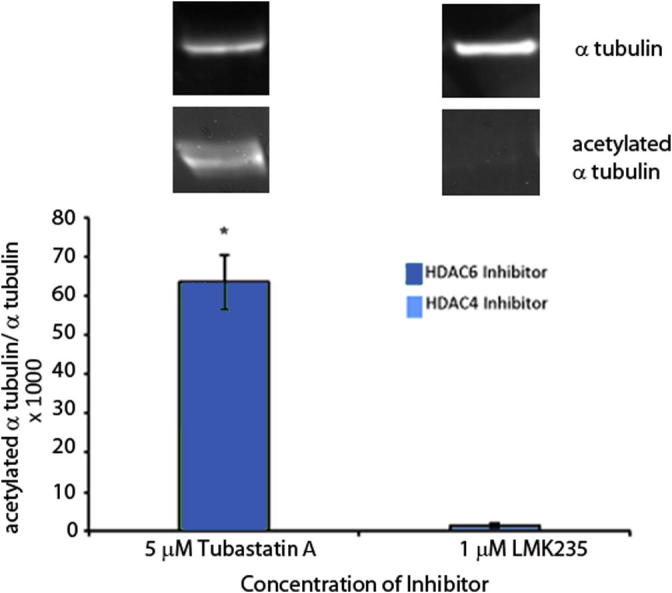
The HDAC4/5 inhibitor LMK235 shows little inhibition of HDAC6 in GT1-7 cells. Cells were treated with 1 μM LMK235 and its effects on inhibition of HDAC6 were determined by measuring deacetylation of α-tubulin by western blotting. The LMK235-treated cells had relatively little acetylated α-tubulin, shown as a ratio of acetylated α-tubulin/total α-tubulin, whereas the HDAC6 inhibitor Tubastatin A (5 μM) significantly increased the amount of acetylated α-tubulin by inhibiting its deacetylation (^∗^p < 0.05).

To study the potential influence of HDACs on NF-κB signaling, the pathway was activated in the presence of HDAC inhibitors. This was explored in cultured primary hypothalamic tanycytes and the well-characterized GT1-7 hypothalamic neuronal cell line. To investigate the influence of HDAC6 in such pathways, cells were treated with the HDAC6-specific inhibitor Tubastatin A (Butler et al., 2010). There is no specific inhibitor for HDAC4 and therefore LMK235 was employed which inhibits the class IIa HDACs 4 and 5, and weakly the class IIb HDAC6 (Marek et al., 2013). To confirm that LMK235 had little cross-over in inhibiting HDAC6 at the 1 μM concentration used in these studies, GT1-7 cells were treated with this concentration of LMK235 and the activity of HDAC6 to deacetylate tubulin was quantified by western blotting. Acetylated α–tubulin was almost undetectable in LMK235-treated GT1-7 cells (Fig. 6 right). In contrast, GT1-7 cells treated with 5 μM Tubastatin A greatly increased the amount of acetylated α–tubulin by inhibiting its deacetylation (Fig. 6 left).

**Fig. 7:**
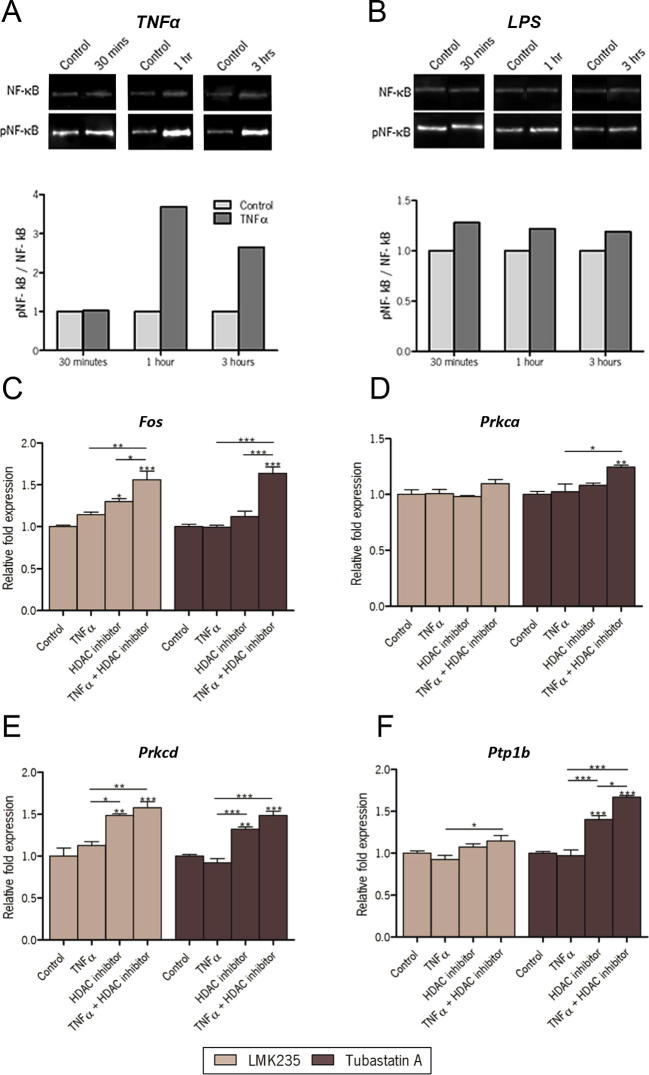
The effect of HDAC inhibitors on NF-κB target gene activation by TNFα in GT1-7 cells. Activation of NF-κB by TNFα (1 ng/mL; A) and LPS (1 μg/mL; B) was compared by western blotting of phosphorylated NF-κB, after 30 min, 1 h and 3 h treatment with the ratio between phosphorylated NF-κB and total NF-κB graphed normalized relative to controls. TNFα showed the greater induction and so the influence of HDAC inhibitors was tested on TNFα induced gene expression. The effect of HDAC4/5 inhibition by LMK235 and HDAC6 inhibition by Tubastatin A was investigated on TNFα-induced expression of (C) *Fos* (D) *Prkca* (E), *Prkcd* and (F) *Ptp1b*. qPCR quantification of gene expression is shown relative to *Actb* levels. ^∗^p < 0.05, ^∗∗^p < 0.01, ^∗∗∗^p < 0.001.

To determine the best activator of NF-κB signaling in GT1-7 cells, a comparison was made between tumour necrosis factor alpha (TNFα) and lipopolysaccharide (LPS). Levels of phosphorylated NF-κB were measured in GT1-7 cells following incubation with TNFα or LPS for 30 minutes, 1 hour or 3 hours (Fig. 7A and B). TNFα induced the greatest increase in NF-κB phosphorylation in GT1-7 cells and hence the effect of HDAC inhibition on rapid induction of gene expression by TNFα (3 h) was investigated. *Fos*, an immediate early gene which encodes one of the components of the activator protein-1 (AP-1) transcription factor, has been described to be regulated by TNFα (Fujioka et al., 2004), but TNFα alone did not induce *Fos* in GT1-7 cells (Fig. 7C). However, inhibition of HDAC4/5 using LMK235 resulted in significant induction of *Fos* by TNFα. Inhibition of HDAC6 by Tubastatin A had a similar effect to allow induction of *Fos* by TNFα (Fig. 7C, left and right). *Prkca*, which encodes protein kinase C alpha, was not induced by TNFα with or without HDAC4/5 inhibition (LMK235) while it was slightly but significantly increased by HDAC6 inhibition (Tubastatin A, Fig. 7D, right). *Prkcd* (protein kinase C delta) was not induced by TNFα but both of the HDAC inhibitors, by themselves, induced *Prkcd* (Fig. 7E, left and right). Finally, *Ptp1b*, a phosphatase acting on the insulin receptor regulated by TNFα via NF-κB in adipose tissue (Zabolotny et al., 2008) and NF-κB in the hypothalamus (Garcia-San Frutos et al., 2012) was investigated. TNFα significantly increased the capacity of HDAC 4/5 inhibitor LMK235 (weakly, Fig. 7F, left) and HDAC6 inhibitor Tubastatin A (to a greater extent, Fig. 7F, right) to induce *Ptp1b.*

**Fig. 8:**
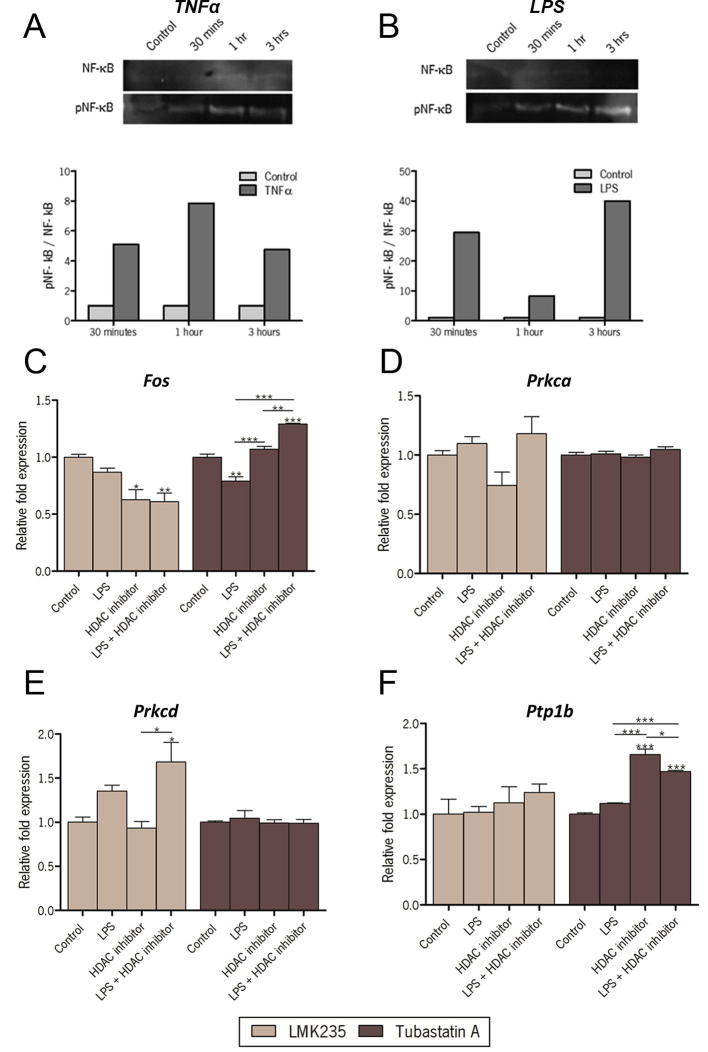
The effect of HDAC inhibitors on NF-κB target gene activation by LPS in tanycytes. Activation of NF-κB by TNFα (1 ng/mL; A) and LPS (1 μg/mL; B) was compared by western blotting of phosphorylated NF-κB, after 30 min, 1 h and 3 h treatment with the ratio between phosphorylated NF-κB and total NF-κB graphed normalized relative to controls. In tanycytes, LPS induced much greater phosphorylation than TNFα and so the influence of HDAC inhibitors was tested on LPS-induced gene expression in tanycytes. The effect of HDAC4/5 inhibition by LMK235 and HDAC6 inhibition by Tubastatin A was investigated on LPS-induced expression of (A) *Fos* (B) *Prkca* (C), *Prkcd* and (D) *Ptp1b*. qPCR quantification of gene expression is shown relative to *Actb* levels. ^∗^p < 0.05, ^∗∗^p < 0.01, ^∗∗∗^p < 0.001.

Tanycytes, a second hypothalamic cell type in which HDACs could function, were also investigated. To determine the best activator of NF-κB signaling in these cells, cultured tanycytes were incubated with TNFα and LPS for 30 min, 1 h or 3 h and the level of phosphorylated NF-κB was quantified (Fig. 8A and B). LPS induced a much greater increase in NF-κB phosphorylation in tanycytes than TNFα and therefore we investigated the effect of HDAC inhibition on rapid induction of gene expression by LPS in tanycytes. *Fos* was weakly repressed by LPS (Fig. 8C) while HDAC4/5 inhibition (LMK235) with or without LPS led to a greater reduction in TNFα (Fig. 8C, left). In contrast, the HDAC6 inhibitor (Tubastatin A) turned LPS into a significant inducer of *Fos* (Fig. 8C, right). Neither the HDAC4/5 nor the HDAC6 inhibitor had a significant effect on LPS induction of *Prkca* (Fig. 8D). However HDAC4/5 inhibition with LMK235 resulted in a significant increase in LPS induction of *Prkcd* expression (Fig. 8E, left)*. Ptp1b*, in contrast, was unaffected by LPS with or without HDAC4/5 inhibition by LMK235 (Fig. 8F, left). However, the HDAC6 inhibitor (Tubastatin A) by itself or with LPS significantly induced *Ptp1b* expression (Fig. 8F, right).

### 3.5 Inhibition of HDACs modifies gene expression induced by nuclear receptor activators

**Fig. 9:**
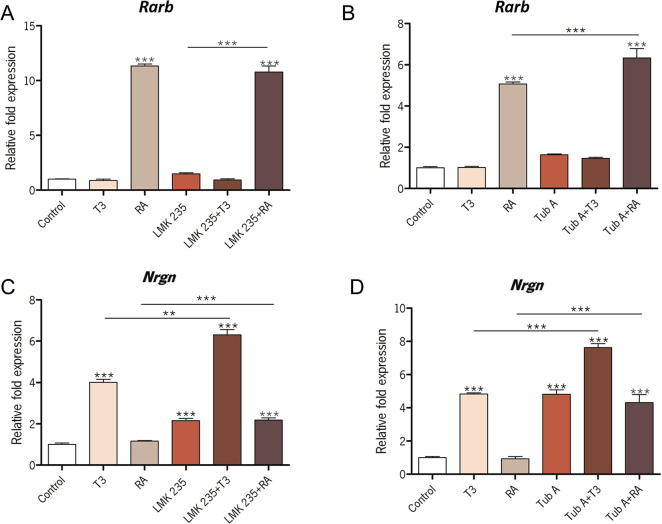
The effect of HDAC inhibitors on nuclear receptor-activated gene expression. The influence of inhibition of (A) HDAC4/5 by LMK235 or (B) HDAC6 by Tubastatin A on induction of *Rarb* by retinoic acid. The influence of inhibition of (C) HDAC4/5 by LMK235 or (D) HDAC6 by Tubastatin A on induction of *Nrgn* by thyroid hormone. qPCR quantification of gene expression is shown relative to *Actb* levels. ^∗∗^p < 0.01, ^∗∗∗^p < 0.001. Tub A, Tubastatin A; RA, retinoic acid; T3, thyroid hormone.

The activity of nuclear receptors is strongly influenced by the level of chromatin condensation, regulated by the acetylation state of histones (McKenna et al., 1999) and this is the case for both the retinoic acid receptor (RAR) and thyroid hormone receptor (THR) (Xu et al., 1999). A seasonal change in HDAC expression would be important for both these nuclear receptors because both RAR and THR mediate signals controlling energy balance and growth in the hypothalamus (Ebling, 2015) while RAR also acts to suppress inflammatory pathways (Kim et al., 2013, van Neerven et al., 2010). Therefore, the effects of the HDAC4/5 inhibitor LMK235 and HDAC6 inhibitor Tubastatin A on the ability of the nuclear receptors RAR and THR to increase transcription in GT1-7 cells was investigated. We assessed the effects of LMK235 and Tubastatin A on expression of *Rarb* and *Nrgn*, genes with well-characterized response elements for RAR (de Thé et al., 1990) and THR (Martinez de Arrieta et al., 1999) respectively, which we had investigated for photoperiodic change (Fig. 1B). After 3 h treatment with retinoic acid the HDAC4/5 inhibitor (LMK235) did not significantly influence the induction of *Rarb* by retinoic acid (Fig. 9A), however HDAC6 inhibition (Tubastatin A) led to an increase in the potency of retinoic acid action on this gene (Fig. 9B). *Rarb* was unresponsive to T3, the bioactive form of thyroid hormone, with or without either of the HDAC inhibitors (Fig. 9A and B). Investigating the effects of T3 on *Nrgn*, both HDAC4/5 and HDAC6 inhibitors significantly increased induction by T3 (Fig. 9C and D). Both inhibitors also resulted in a significant increase in the background expression of *Nrgn* (Fig. 9C and D). Retinoic acid had no significant effect on the expression of *Nrgn*, either in the presence or absence of the HDAC inhibitors (Fig. 9C and D).

## 4 Discussion

The photoperiodic F344 rat provides a unique rat model to study hypothalamic regulation of appetite and energy homeostasis using a natural environmental switch to force a homeostatic change rather than more extreme manipulations, such as high-fat diet. This study focused on possible epigenetic changes that may be part of this seasonal switch, examining alterations in gene expression of HDACs, which deacetylate histones, promoting chromatin condensation and thus suppressing gene expression. Seasonal epigenetic changes have been shown in the immune system (Stevenson et al., 2014) and epigenetic alterations in the brain can have profound effects on behavior (Mathews and Janusek, 2011). Three members of the HDAC family, *Hdac*4, 6 and 9, were found to be under photoperiodic control and were expressed at higher levels under long-day (summer-like) versus short-day (winter-like) conditions. For *Hdac4*, these changes in mRNA expression were evident in the ependymal cells and tanycytes lining the third ventricle and when measured specifically in these cells, the change in gene expression was large (5-fold). TSH was shown to induce *Hdac4* expression both *in vivo* and *ex vivo* and therefore TSH is likely to act as an intermediary by which melatonin may trigger seasonal changes in *Hdac4* expression, as is the case for a number of other genes which are key intermediaries in melatonin’s control of seasonal change in the hypothalamus (Dardente et al., 2014). In contrast, *Hdac6* was not inducible by TSH in the hypothalamus and its seasonal change is presumably mediated by alternative factors.

To study the potential functions of these HDACs, chemical inhibitors were used on cultured primary hypothalamic tanycytes and a representative hypothalamic neuronal cell line, GT1-7. Given the primarily nuclear location of these HDACs, nuclear signaling pathways were investigated. The initial focus was on NF-κB, of particular interest given the change in *Nfkb1* gene expression between seasons; this is the second inflammatory mediator shown to change its expression with photoperiod, the other being chemerin (Helfer et al., 2016). The concept that the hypothalamus in photoperiodic animals may respond differently to inflammatory signals depending on day length was proposed several years ago (Fonken et al., 2012). The use of HDAC inhibitors, one primarily acting on HDAC4/5 and another specific for HDAC6, suggested that these HDACs act to reduce the capacity of the factors that signal via NF-κB, TNFα- and LPS-activated pathways, to regulate genes acting on several pathways including AP-1 (Fos), protein kinase C and the protein-tyrosine phosphatase Ptp1b. The decrease in *Hdac4, 6* and *9* during short-day photoperiod, and presumed decline in their corresponding protein, will increase the capacity of NF-κB to induce genes such as *Fos, Prkca* and *Ptp1b*. This coincides with the increase in *Nfkb1* under the same short-day conditions and the combination of decreased *Hdac* expression and increased NF-κB may potentiate the ability of this inflammatory signal to regulate gene expression during short-day photoperiod.

TNFα and LPS are proinflammatory molecules and the concept that the HDACs are involved in inflammatory processes has been previously suggested by the requirement for HDAC4 in efficient inflammatory cytokine production activated by LPS (Wang et al., 2014). Prolonged LPS treatment leads to HDAC4 degradation via caspase 3 (Wang et al., 2014). In macrophages, HDAC4, when activated by cAMP, shuttles to the nucleus where it inhibits NF-κB activity on proinflammatory genes (Luan et al., 2014). In adipose tissue, in which inflammation is proposed to be part of the trigger for obesity, HDAC4 is known to inhibit NF-κB activity in the nucleus (Abu-Farha et al., 2013, Luan et al., 2014). Further suggesting the existence of inflammatory signaling pathways in the hypothalamus, LPS-induced inflammation in hypothalamic tanycytes leads to an increase in the capacity of thyroid hormone to induce *Dio2* via an NF-κB mediated pathway (Fekete et al., 2004).

Both HDAC4 and 6 shuttle between the nucleus and cytoplasm, but much attention has been directed towards their function in the cytoplasm. HDAC6 deacetylates several cytoplasmic proteins including tubulin and HSP90 (Yang et al., 2013) while there is the suggestion that HDAC4 may not function as a lysine deacetylase (Mielcarek et al., 2013). However, both HDAC4 and HDAC6 can be found in the nucleus under some circumstances (Fitzsimons, 2015, Liu et al., 2012) and the results of our immunohistochemistry support this. HDAC4 can repress transcription (Wang et al., 1999) and may recruit other histone deacetylases in this action (Chan et al., 2003, Zhang et al., 2002).

This repressive action of HDACs on gene expression can have a strong effect on nuclear receptor-induced transcription and our studies pointed to such a repressive action of HDACs on target genes of the nuclear receptors RAR and THR. Both thyroid hormone (Dardente et al., 2014) and retinoic acid (Shearer et al., 2012b) act to regulate gene expression during LD photoperiod and a simultaneous increase in HDAC in LD conditions will have a limiting effect on these signaling pathways. This repressive action of HDACs has been proposed, for instance, in HDAC4’s association with reduced binding of THR to the thyroid hormone response element (TRE) of *Glut4* (Raychaudhuri et al., 2014). Movement of HDAC4 between cytoplasm and nucleus is controlled by its phosphorylation (Fitzsimons, 2015). In the case of HDAC6, it is acetylation that controls movement between nucleus and cytoplasm (Liu et al., 2012). HDAC6 can be acetylated by the nuclear co-activator and acetyltransferase, p300 (Han et al., 2009). Reciprocally, it has been proposed that HDAC6 acts to modulate p300-dependent transcription by binding to sumoylated p300 (Girdwood et al., 2003). HDAC6 may also be involved in repression of p21 expression by Runx2 (Westendorf et al., 2002) and the repressive action of NF-κB on particular genes (Zhang and Kone, 2002). HDAC6 has been found to be associated with other nuclear receptors including the vitamin D receptor (Toropainen et al., 2010). HDAC6 is also a nuclear cofactor for ligand-dependent corepressor (LCoR) in its suppression of estrogen-induced transcription (Palijan et al., 2009).

The importance of epigenetic control of hypothalamic gene regulation has been increasingly recognized. For instance, the hypothalamic response to dietary change, whether fasting or high-fat diet, includes a change in the pattern of *Hdac* expression (Funato et al., 2011). Changes in the environment of a mother can result in long-term behavioral changes in her offspring via epigenetic reprogramming of the hypothalamus (Gali Ramamoorthy et al., 2015), while HDACs help guide the sexual differentiation of the hypothalamus during development of the brain (Matsuda et al., 2012)**.** Our findings of a switch in *Hdac* expression driven by a change in photoperiod may not be unexpected and part of the mechanism involved in the seasonal switch in control of physiology and behavior.

Our results suggest that increased HDAC4 and 6 during LD (summer-like) conditions will limit the capacity of inflammatory factors acting through NF-κB to induce gene expression, as well as decrease the ability of thyroid hormone and retinoic acid to induce gene expression. This occurs at a time when NF-κB is also falling, but when other factors are on the rise; during LD there is an increase in the inflammatory factor chemerin (Helfer et al., 2016) and both thyroid hormone (Ross et al., 2011) and retinoic acid (Helfer et al., 2012). This indicates that inflammation as a general phenomenon does not simply rise and fall between SD and LD but, as may be expected, different elements of the inflammatory pathway are used as molecular steps in both SD and LD. As such, a general induction of inflammation in the hypothalamus, for instance from infection, would not be expected to induce a simple photoperiodic switch but would have aspects of both SD and LD in cell signaling and perhaps physiology. It would also be predicted that photoperiod will influence the impact of inflammation on hypothalamic function, and these ideas will be intriguing to test in the future. The inhibitory action of HDAC4 and 6 during LD when thyroid hormone and retinoic acid signaling increase suggest that there are subsets of genes in particular hypothalamic regions, or times during LD, in which the action of these nuclear receptor ligands require inhibition. The next stage of investigation into the photoperiodic switch in HDAC expression will explore this. Something similar is seen during LD for retinoic acid in which there is a large increase in the retinoic acid catabolic enzyme Cyp26b1 during LD conditions (Helfer et al., 2012) which would oppose the simultaneous increase in retinoic acid signaling (Shearer et al., 2010). A limitation of the study to note is that the mechanistic study of HDAC function is performed *in vitro* rather than *in vivo* and future studies of *Hdac4* and *Hdac6* knockouts will be valuable when performed in photoperiodic animals, which is now achievable through CRISPR-Cas9-based approaches.

The types of systems we have shown to be controlled by HDACs in the hypothalamus, NF-κB-mediated inflammatory pathways, and activation of gene expression by thyroid hormone and retinoic acid, have been described to change in multiple animals that photoperiodically switch in their physiology (Ebling, 2015, Helfer et al., 2016). A change in hypothalamic HDAC expression would be of great interest to study in these species. A question arises whether similar alterations might occur in the human hypothalamus, the question hinging on whether human physiology has a seasonally-driven rhythm. Studies have derived varied conclusions (Foster and Kreitzman, 2010). Disease susceptibility (Stevenson et al., 2015), immune function and metabolic disease (Dopico et al., 2015, Goldinger et al., 2015), as well as psychiatric disease and seasonal affective disorder (Magnusson and Boivin, 2003, Qin et al., 2015) have all been shown to change with season. The system that controls photoperiodism i.e. the night time rise in melatonin, is known to function in humans. Further, seasonal changes in the morphology of the human hypothalamus have been reported (Hofman and Swaab, 1992). Thus the control system we describe in the rat may be relevant to parallel regulatory systems in the human hypothalamus.

Our results point to a potential role for HDACs in both inflammatory signaling (via NF-κB) and hormonal (thyroid hormone and retinoic acid) pathways known to act in the hypothalamus (Purkayastha and Cai, 2013, Valdearcos et al., 2015) and HDAC4 and 6 may sit at a nexus linking epigenetic modulation of hormone and inflammatory pathways in the hypothalamus. NF-κB is described as a pro-inflammatory master switch determining production of various inflammatory mediators, triggering hypothalamic inflammation that can lead to altered hypothalamic control of metabolism to result in metabolic syndrome (Purkayastha et al., 2011, Zhang et al., 2008). Thyroid hormone has control over many hypothalamic roles from appetite to reproductive function (Lechan and Fekete, 2005) while retinoic acid is likely to supplement the function of thyroid hormone (Stoney et al., 2016b) as well as controlling cell behavior such as hypothalamic neurogenesis (Shearer et al., 2012c). HDAC expression is an epigenetic switch that can cascade onto these many pathways through repression of NF-κB, thyroid hormone and retinoic acid control of hypothalamic gene expression.
